# Artificial Intelligence in Postmenopausal Health: From Risk Prediction to Holistic Care

**DOI:** 10.3390/jcm14217651

**Published:** 2025-10-28

**Authors:** Gianeshwaree Alias Rachna Panjwani, Srivarshini Maddukuri, Rabiah Aslam Ansari, Samiksha Jain, Manisha Chavan, Naga Sai Akhil Reddy Gogula, Gayathri Yerrapragada, Poonguzhali Elangovan, Mohammed Naveed Shariff, Thangeswaran Natarajan, Jayarajasekaran Janarthanan, Shiva Sankari Karrupiah, Keerthy Gopalakrishnan, Divyanshi Sood, Shivaram P. Arunachalam

**Affiliations:** 1 Digital Engineering & Artificial Intelligence Laboratory (DEAL), Mayo Clinic, Jacksonville, FL 32224, USA; rachnakukreja7@gmail.com (G.A.R.P.); samiksha190404@gmail.com (S.J.);; 2 Roxborough Memorial Hospital, Philadelphia, PA 19128, USA; varshini.maddukuri@gmail.com; 3 Duke Molecular Physiology Institute, Duke University, NC 27708, USA; 4 Department of Internal Medicine, Wright Medical Center, Scranton, PA 18503, USA; 5 Department of Internal Medicine, UCHealth Parkview Medical Center, Pueblo, CO 81003, USA; 6 Division of Pulmonology, Department of Critical Care Medicine, Mayo Clinic, Jacksonville, FL 32224, USA

**Keywords:** menopause, postmenopausal complications, artificial intelligence, machine learning, risk prediction, telemedicine, health equity, wearable sensors, mental health, estrogen

## Abstract

**Background/Objectives**: Menopause, marked by permanent cessation of menstruation, is a universal transition associated with vasomotor, genitourinary, psychological, and metabolic changes. These conditions significantly affect health-related quality of life (HRQoL) and increase the risk of chronic diseases. Despite their impact, timely diagnosis and individualized management are often limited by delayed care, fragmented health systems, and cultural barriers. **Methods**: This review summarizes current applications of artificial intelligence (AI) in postmenopausal health, focusing on risk prediction, early detection, and personalized treatment. Evidence was compiled from studies using biomarkers, imaging, wearable sensors, electronic health records, natural language processing, and digital health platforms. **Results**: AI enhances disease prediction and diagnosis, including improved accuracy in breast cancer and osteoporosis screening through imaging analysis, and cardiovascular risk stratification via machine learning models. Wearable devices and natural language processing enable real-time monitoring of underreported symptoms such as hot flushes and mood disorders. Digital technologies further support individualized interventions, including lifestyle modification and optimized medication regimens. By improving access to telemedicine and reducing bias, AI also has the potential to narrow healthcare disparities. **Conclusions**: AI can transform postmenopausal care from reactive to proactive, offering personalized strategies that improve outcomes and quality of life. However, challenges remain, including algorithmic bias, data privacy, and clinical implementation. Ethical frameworks and interdisciplinary collaboration among clinicians, data scientists, and policymakers are essential for safe and equitable adoption.

## 1. Introduction

Menopause is defined as the permanent cessation of the menstrual cycle, diagnosed after at least one consecutive year of amenorrhea, without any obvious physiological or pathological cause such as pregnancy or lactation. The mean age at which menopause occurs is 51 [1,2]. Worldwide, around 47 million women transition into menopause each year. The global population of menopausal women is expected to rise quickly from 467 million in 1990 to 1.2 billion by 2030. Between 1990 and 2030, the number of postmenopausal women will triple in the developing world, accounting for most of this rise. Additionally, the percentage of postmenopausal women in the population will rise from 9% in 1990 to 14% in 2030 [3].

The underlying biological mechanism is the depletion of ovarian follicles, which leads to a drastic and sustained decrease in estrogen levels unless hormone replacement therapy is initiated [4]. This endocrine change underlies both the immediate symptoms and long-term health implications of menopause. The primary source of estrogens after menopause is the peripheral conversion in adipose tissue of adrenal androgen precursors, particularly androstenedione, to estrogens, mostly estrone. Therefore, body fat is a key regulator of estrogen levels in postmenopausal women [5,6].

Epidemiological studies demonstrate that lifestyle and demographic factors such as smoking may influence the timing of menopause, but the health burden is largely driven by estrogen deficiency rather than age alone [5,6,7,8,9,10]. According to NICE guidelines, menopause is generally a clinical diagnosis in women over 45, without the need for routine laboratory testing, except in atypical or premature cases [11,12].

Despite the broad range of menopausal complications, relatively few studies have been performed on the barriers to access to menopausal treatment [13]. Immediate symptoms and the long-term health implications of menopause are significant, yet the availability and education of menopausal care remain strikingly limited [14]. In populations such as low- and middle-income countries (LMICs), access to medical practitioners with specialized expertise in menopause is especially limited [13,14]. In many cultures, menopause has long been considered a taboo subject, and women are reluctant to bring up the subject [15]. More than 60% of women going through menopause struggle to obtain proper treatment [16], and only 54% of them seek medical advice for menopausal symptoms [17]. A survey by the Mayo Clinic found that a large number of medical residents lack the necessary training in managing menopause, particularly in prescribing hormone therapy [18]. Additionally, the cultural and language barriers contribute to the miscommunication between patients and providers, leading to underdiagnosis of problems. The outrageous medical costs, the absence of health insurance, and the geographic restrictions on accessing hospitals are other significant challenges to accessible medical care [19]. Furthermore, many medical professionals around the world utilize indices; for instance, hospitals in China frequently utilize the Kupperman Menopausal Index (KMI) to assess the severity of menopausal symptoms, which takes up a considerable amount of time, delaying diagnosis and management [20]. These unmet needs emphasized that menopause education has to be integrative and holistic, including healthcare workers, supportive policies, and available services to assist women in this stage to be both healthy and informed [21].

### Impact on Quality of Life

Beyond physical changes, the menopausal transition is associated with psychological, cognitive, and social challenges that collectively impair health-related quality of life (HRQoL) [10,11]. Importantly, barriers such as cultural stigma, limited provider training, high costs, and fragmented healthcare systems contribute to widespread underdiagnosis and under treatment [13,14,15,16,17,18,19]. Even where indices such as the Kupperman Menopausal Index are used, their time-consuming nature can delay timely management [20]. These unmet needs highlight the importance of approaches that can improve efficiency, accuracy, and personalization in postmenopausal care [21,22]. Artificial intelligence (AI) offers a potential solution by bridging these gaps. AI-driven approaches can detect symptom patterns, integrate clinical and lifestyle data, and provide personalized treatment recommendations [23,24]. By analyzing complex health information, including biomarkers, imaging, electronic health records, and wearable sensor outputs, AI models can support early identification, risk stratification, and targeted interventions [25]. Importantly, AI-enabled platforms can democratize access to menopausal care through telemedicine and digital health solutions, thereby helping to mitigate inequities [25].

Therefore, the purpose of this review is to evaluate the role of AI in risk prediction, early diagnosis, monitoring, and treatment of postmenopausal health issues. Beyond summarizing current applications, this review emphasizes critical gaps, including algorithmic bias, data privacy, and limited clinical integration, while highlighting the broader potential of AI to transform postmenopausal care into a proactive and holistic model.

## 2. Materials and Methods

### 2.1. Inclusion Criteria

Population: Studies involving premenopausal, perimenopausal, or postmenopausal women thatfocus on the early detection or management of menopausal health issues.

Intervention/Technology: Research utilizing Artificial Intelligence (AI)—including Machine Learning (ML), Deep Learning (DL), or comparable computational approaches; for risk prediction, diagnosis, monitoring, or management of menopausal health concerns.

Scope of Study: Studies must focus on AI-driven models or tools addressing major complications (e.g., cardiovascular disease, osteoporosis) or minor complications (e.g., vasomotor symptoms, mental health challenges), with particular attention to early detection during the premenopausal or menopausal transition stages.

Study Types: Peer-reviewed original research, systematic reviews, meta-analyses, or narrative reviews that present AI-based approaches relevant to menopausal care.

Data & Technique Transparency: Only studies that clearly describe AI methodologies, datasets, validation strategies, and reported results were included.

Addressing Heterogeneity: To account for the diversity in AI literature, studies were categorized by AI type (ML, DL, NLP, etc.), outcome focus (major vs. minor complications), and validation strategy. This approach ensured consistent evaluation and meaningful comparison across studies.

### 2.2. Exclusion Criteria

- Studies not employing AI or computational models, including standard clinical trials.
- Animal or in vitro studies.
- Conference abstracts, editorials, commentaries, or opinion pieces without original AI data or analysis.
- Studies published before 2010, unless they were foundational AI papers directly relevant to menopausal care.

### 2.3. Literature Search Strategy

A comprehensive literature search was conducted across PubMed, Scopus, and IEEE Xplore databases to identify studies published between January 2010 and August 2024. Search terms combined both controlled vocabulary and free-text keywords related to menopause (“menopause,” “postmenopausal,”“perimenopausal”) and artificial intelligence (“machine learning,” “deep learning,” “neural networks,” “predictive modeling” “natural language processing”). Reference lists of relevant reviews were also screened to ensure inclusion of additional studies. After removing duplicates, titles and abstracts were reviewed to determine relevance. Full texts were then assessed independently by all reviewers based on predefined inclusion and exclusion criteria, focusing on AI-driven applications for postmenopausal health. Discrepancies were resolved through discussion. A total of 38 studies were finally included for synthesis.

## 3. Postmenopausal Complications: A Spectrum of Major Complications

### 3.1. Cardiovascular Disease: Pathophysiology

The transition into menopause in a woman’s life is marked by a significant hormonal imbalance, notably due to falling estrogen levels [3]. These changes extend beyond the reproductive system, impacting various physiological systems. Among these, cardiovascular (CV) health is significantly affected, with an increased risk of heart-related complications emerging post-menopause [26,27,28]. Numerous studies have rigorously investigated the association between the timing of menopause and CV outcomes [14,29,30,31,32,33]. A meta-analysis by Atsma et al. (2006) [29] reported that women who had bilateral oophorectomy were 4.55 times more likely to develop cardiovascular disease than women who did not, while early menopause conferred an RR of 1.25. Wellons et al. (2012) [34] found similar results in the Multi-Ethnic Study of Atherosclerosis (MESA). They reported that the women with a history of early menopause had markedly worse coronary heart disease (CHD) outcomes and shorter stroke-free survival, even after correcting for age and race/ethnicity. The protective role of estrogen and progesterone on the cardiovascular system is well-established. Estrogen, for example, helps to maintain lower levels of total cholesterol and low-density lipoprotein (LDL) cholesterol. The decline in estrogen levels alleviates this protective effect, potentially increasing the risk of cardiovascular diseases (CVD) post menopause [3]. Several interconnected factors contribute to the elevated cardiovascular risk observed in postmenopausal women. These include alterations in metabolic and endocrine processes and the direct consequences of estrogen deprivation on vascular function. Estrogen is essential for preserving vascular health through many mechanisms, including promoting vasodilation, exhibiting anti-inflammatory actions, offering antioxidant properties, and regulating neuroendocrine function. These defenses are compromised by the drop in estrogen levels that occurs with menopause [35,36]. Epidemiological data further underscore the impact of menopause on cardiovascular risk. The mortality rates of females compared to males from CVD were found to be higher after the age of 50, demonstrating that the protective effect of reproductive life in a woman against CVD may decrease following menopause [3]. In populations with higher mortality rates, women are exposed to greater risks at first, which decline to a slight advantage at the age of 60 and then disappear at old age. In populations with a low mortality rate, women exhibit a U-shaped growth, having a small advantage in the initial reproductive years that reaches a peak around 50 years of age and afterward declines to almost equal. Such observances further support the protective effect of reproductive life [3]. CVD stands as a major global health concern for women in the postmenopausal phase of life and is recognized as the leading cause of death among women in the developed world [37]. Menopause leads to altered fat distribution in the body, causing a rise in visceral adipose tissue, or heart fat, that collects around the abdominal organs and is associated with a higher risk of CVD compared to subcutaneous fat [26]. This can be explained by a shift in body fat distribution from a gynoid (pear-shaped) to an android (apple-shaped) pattern [38]. The visceral fat is metabolically active and releases several inflammatory mediators and adipokines, contributing to insulin resistance, dyslipidemia (abnormal lipid levels), metabolic syndrome, and an overall heightened risk of heart-related complications [26,38,39]. There is also an increased prevalence of central obesity (measured by waist-hip ratio) in postmenopausal women, which is another independent risk factor for CVD [40]. The body’s coagulation system also changes post-menopause. Levels of antithrombin III, factor VII coagulant activity (VIIc), and plasma fibrinogen, all crucial elements in blood clotting, are significantly elevated after menopause [41]. Various other factors that contribute to the increased risk are glucose intolerance, impaired lipid levels, increased BP, increased response to the sympathetic nervous system, impaired endothelial system, and vascular inflammation [21]. Intriguingly, studies have also identified a link between menopausal symptoms, particularly vasomotor symptoms (VMS) such as hot flashes, and CV risk. Regardless of menopausal state, increasing systolic BP has been linked to hot flash frequency. VMS may be an early indicator of endothelial dysfunction and a predictor of future cardiovascular risk because it is more common in late postmenopausal women with a hypertension diagnosis. Vascular response to endothelium-mediated dilatation is inversely correlated with the severity of VMS, indicating a reduction in endothelial function and vascular compliance. A procoagulant hemostatic and unfavorable hemodynamic profile, which includes a poorer overall heart index and stroke volume index, is also linked to VMS. According to these results, women who experience myocardial infarction (MI) and VMS during the menopausal transition are more likely to die from CVD and have worse endothelial function than those who experience VMS later in menopause [36]. Some of the cardiac complications related to menopause are as follows:

#### 3.1.1. Atherosclerosis

One major change during menopause is the shift in lipid levels. There is a tendency for total cholesterol, apolipoprotein B (APOB), triglycerides, and LDL cholesterol (bad cholesterol) levels to rise, while the anti-atherogenic effect of HDL cholesterol (good cholesterol) seems to decline [26,36,42]. The vasculature also suffers as a result of insulin resistance, which leads to smooth muscle cell proliferation, vasoconstriction, and increased pro-inflammatory activity [7]. Estrogen normally helps to maintain lower serum fibrinogen levels and higher prostacyclin levels, both of which contribute to reducing the risk of thrombosis (blood clot formation) [42]. Furthermore, a decline in estrogen levels causes a rise in oxidative stress, characterized by overproduction of free radicals and decreased antioxidants [43]. These alterations work together to cause atherosclerosis, a disorder that causes arteries to stiffen and narrow, raising the risk of cardiovascular events. Moreover, menopause-related sleep problems have also been connected to CV health. A higher degree of aortic calcification and increased carotid atherosclerosis are linked to worse sleep quality and shorter sleep duration [26]. This postmenopausal increase in atherosclerosis directly elevates the risk of coronary artery disease (CAD) [43]. Interestingly, estrogen supplementation has been shown to enhance coronary blood flow, decrease coronary resistance, and increase coronary cross-sectional area in postmenopausal women undergoing cardiac catheterization [41]. Studies have revealed a significantly higher prevalence of CAD in postmenopausal women compared to premenopausal women [40]. Notably, the epicardial adipose tissue (EAT) and paracardial adipose tissue (PAT), types of fat surrounding the heart that are considered to have a more detrimental impact than general visceral fat and are emerging risk factors for CAD, also increase after menopause. A study by the SWAN (Study of Women’s Health Across the Nation) cardiovascular fat ancillary research found that late Perimenopausal/postmenopausal women had 9.9% more EAT and 20.7% more PAT than premenopausal women [43].

#### 3.1.2. Angina

Research has indicated a correlation between the age at which menopause occurs and the risk and severity of angina following an MI. Women who had menopause before the age of 40, either naturally or surgically, had twice the risk of angina and experienced severe angina post-MI compared to those who transitioned into menopause at or after the age of 50 [44].

#### 3.1.3. Hypertension

Menopause also alters the blood pressure (BP) regulation in the body. Before menopause, women typically have lower BP than men of the same age; however, this advantage diminishes as women transition through menopause. Postmenopausal women are more susceptible to hypertension, in part because of their heightened sensitivity to salt, which almost doubles in 4 months [26]. Estrogen has demonstrated the ability to suppress the paradoxical vasoconstriction that occurs in atherosclerotic coronary arteries in response to acetylcholine, indicating that it may have alpha2-inhibiting and calcium channel-blocking effects [42]. Additionally, estrogen deficiency impacts the endothelial function, leading to a decline in the release of cardioprotective and vasodilatory nitric oxide, which leads to vasoconstriction and an increase in endothelin, a strong vasoconstrictor. This hormonal shift also stimulates the renin–angiotensin–aldosterone system (RAAS), both of which can contribute to elevated blood pressure [36]. This vascular aging caused by menopause, characterized by constricted vessels, appears to be more accelerated than the physiological vascular aging [43]. Moreover, insulin resistance can cause pancreatic beta cells to secrete excessive insulin, negatively impacting the vasculature. These side effects, which include fluid and salt retention and vasoconstriction, can collectively lead to hypertension [7]. 

#### 3.1.4. Heart Failure

The various side effects, such as a rise in salt sensitivity and vasoconstriction, increase the strain on the heart, with an increased risk of heart failure [38]. Estrogen also plays a role in maintaining healthy cardiovascular biomechanics, influencing both relaxation and contractility of the ventricles. Hence, declining estrogen levels post-menopause come with an increased risk of heart failure [42]. Studies have also demonstrated a link between early menopause and increased risk of heart failure. According to the Multi-Ethnic Study of Atherosclerosis, women who underwent menopause before the age of 45 had almost more than double the risk of heart failure compared to those who underwent menopause later. According to another study, women who had a natural menopause between the ages of 40 and 45 had a 36% higher incidence of heart failure than women who experienced menopause between the ages of 50 and 54, with their risk decreasing by 2% for each year their age at menopause increased [44].

#### 3.1.5. Stroke

Several studies have shown an association between early-onset menopause or a shorter duration of ovarian activity and a higher risk of stroke, particularly ischemic stroke [44]. High blood pressure, which is one of the leading risk factors for stroke, also increases after menopause, thereby increasing the chances of stroke along with it [38]. For women aged 40 to 89, the risk of stroke death doubles for every 20 mmHg rise in systolic and 10 mmHg increase in diastolic blood pressure [45]. Postmenopausal women also have higher levels of fibrinogen, a protein that aids in blood coagulation. Elevated fibrinogen levels also raise the risk of thrombotic events like strokes by facilitating the development of clots [46]. Furthermore, the various risks associated with menopause, including endothelial dysfunction, oxidative stress, and dyslipidemia, contribute to the deposition of lipids in the arterial walls, leading to the formation of atherosclerotic plaques, further increasing the risk of stroke [39].

#### 3.1.6. Palpitations

Palpitations, defined as an uneasy awareness of an abnormal heartbeat, can occur in women of all age groups, particularly during the luteal phase of the menstrual cycle, pregnancy, and the menopausal period. In Perimenopausal women, palpitations are frequently benign and appear to be related to increased sympathetic activity due to menopause [5].

#### 3.1.7. Screening, Prevention, and Management

Given the increased CV risk in postmenopausal women, regular screening is crucial. A comprehensive screening panel should include regular BP, lipid levels, inflammatory markers, and BMI measurements. Assessment of a family history of heart disease and strokes is also important. The 10-year risk of myocardial infarction is determined using risk assessment techniques such as the Framingham model and the American Heart Association model, which take into account variables including age, sex, race, blood pressure, cholesterol, diabetes, and smoking status [37]. Several lifestyle and medical interventions are employed to minimize CVD risk in postmenopausal women. These include smoking cessation, weight management through a balanced diet and regular exercise, management of high blood pressure, and treatments for elevated cholesterol and thrombosis risk [37]. Most of the risks stem from declining estrogen levels, which not only directly affect CV function and metabolism but also exaggerate classic risk factors of CVD like body fat distribution, glucose tolerance, plasma lipids, blood pressure, sympathetic tone, and endothelial function [38]. Current guidelines recommend the use of statins for both primary and secondary prevention of cardiovascular events in women at high risk for CVD [45]. Hormone replacement therapy (HRT) is also considered for its potential cardioprotective effects in postmenopausal women [38]. HRT has been shown to positively influence various CVD risk factors, such as improving lipid profiles, reducing visceral adiposity, improving insulin sensitivity, and having beneficial effects on BP [39]. Observational studies have suggested that postmenopausal women who took estrogen had consistently lower rates of events than those who did not, according to observational studies investigating the relationship between unopposed postmenopausal estrogen usage and risk of coronary heart disease events [1]. However, HRT has a complex relationship with cardiac health. The timing, dose, and route of administration play a crucial role in determining its overall effects, influencing whether it is beneficial or potentially harmful. For instance, it has a beneficial effect in early atherogenesis by improving plasma lipids, regulating endothelial function, and increasing NO levels, whereas in already established atherosclerosis, it can increase matrix metalloproteinase (MMP) expression, potentially leading to plaque instability and rupture [45]. Therefore, a careful evaluation of the route of administration, dosage, and individual underlying risk factors is essential before initiating HRT [27].

### 3.2. Cancers

#### 3.2.1. Breast Cancer

Breast cancer, which is the leading type of cancer affecting women worldwide, is closely linked to hormonal factors. Menopause and its hormonal variations affect the overall health of women and are an important time for women to evaluate the risk for breast cancer [47]. Studies have shown that women who underwent surgically induced menopause had a 60% lower risk of breast cancer than those who went through natural menopause between the ages of 45 and 54 [48]. This protective effect of surgically induced menopause is particularly prominent at younger ages. The most pronounced reduction in risk is observed in women who had surgical menopause before age 35, and the benefit remains significant up to age 50. On the other hand, there was a positive relationship between the age of naturally induced menopause and breast cancer risk. The pattern implied that women who go through the natural menopause later in life could be more likely to have breast cancer than people who go through it sooner. A study found that the risk rose by a factor of 1.029 for each year that the woman was older at menopause [49]. These results highlight the intricate relationship between menopausal hormones and breast cancer development [48]. A study by Berrino et al. demonstrated that both elevated levels of serum testosterone and estradiol are associated with an increased breast cancer risk in postmenopausal women, with estradiol having a much bigger effect [50]. Paffenbarger et al. revealed several factors elevating breast cancer risk, including higher education levels, lower parity, early menarche, late childbirth, delayed menopause, and a family history of breast cancer [51]. Mammographic density has also emerged as an independent predictor of breast cancer risk, with impact across both pre- and post-menopause, signifying the importance of including mammographic density in personalized screening, especially for women undergoing or past menopause [52]. Endogenous ovarian hormones were found to have a bigger impact on estrogen receptor-positive and lobular breast cancers compared to estrogen receptor-negative and ductal cancers [49]. Current or recent use of HRT was shown to modestly increase breast cancer risk, with a greater risk associated with longer duration of use and decreasing with the cessation of usage [53]. Postmenopausal women who were on HRT exhibited a higher incidence of estrogen receptor-positive and lobular breast cancers compared to estrogen receptor-negative and ductal cancers [49]. Further, estrogen-blocking treatments were observed to improve survival for estrogen receptor-positive but not for estrogen receptor-negative breast cancer [49]. A collective influence of genetic, hormonal, and dietary factors leads to the development of breast cancer. Among the postmenopausal women, the increased adipose tissue raises local estradiol via aromatase, supporting tumor growth. High-fat diets are associated with the accumulation of cholesterol and the development of insulin resistance, which are both linked to breast cancer. Obesity further aggravates the risk of breast cancer post menopause [47]. Interestingly, while HRT is considered for symptomatic relief of menopausal symptoms, its usage is quite a subject of debate due to the possible association with breast cancer [54]. A meta-analysis by Reeves et al. demonstrated that the risk of breast cancer is higher in women using combined estrogen-progestin therapy (EPT) as opposed to those using estrogen-only therapy (EOT). The risk declined after stopping HRT and was modulated by BMI. HRT use was associated with the increased risk of breast cancer, particularly of lobular and tubular histological types [55]. Current smoking status was also observed to have a link with breast cancer [56]. Interestingly, while menopause affects breast cancer risk, conversely, the adjuvant therapies for breast cancer, including chemotherapy and endocrine therapies like tamoxifen or aromatase inhibitors, were also linked to premature menopause [57]. Longer reproductive life poses a significant risk factor for breast cancer, with early menarche associated with a higher risk compared to late menopause, possibly due to heightened estrogen sensitivity during pubertal breast development [58]. In summary, while the genetic factors and early reproductive events substantially influence breast cancer risk, the lifestyle changes post menopause, for example, maintaining a healthy weight, being physically active, minimizing alcohol intake, and managing hormone therapy effectively, can still significantly decrease the disease risk. This underlines the importance of public health strategies directed toward modifiable lifestyles in postmenopausal women [59]. 

#### 3.2.2. Ovarian Cancer

Ovarian cancer, another malignancy with increased incidence in postmenopausal women, is influenced by both hormones and dietary trends [47]. Almost 85–90% of all ovarian cancers are diagnosed after menopause [60]. The cells on the surface of the ovary, called ovarian surface epithelial cells, go through replication due to the predominant action of estrogen during the menstrual cycle. Defective control of this process may result in cysts or tumors, for instance, surface epithelial tumors. Consumption of a high-fat diet produces more estrogen, which promotes proliferation in the female urinary tract, while a diet that is rich in animal protein can be responsible for the entrance of outside estrogens that have a carcinogenic effect [47]. Studies have found that surgically induced menopause, particularly via hysterectomy alone, was associated with an approximately 30% lower risk of ovarian cancer development, while natural menopause did not affect subsequent ovarian cancer development. The age of menopause also did not appear to be a significant risk factor for the development of ovarian cancer. This implies that the natural hormonal fluctuations during the aging process and the lack of ovarian function are not directly linked to the risk of developing ovarian cancer. OCP use has been linked with a lower risk due to suppression of ovulation, thereby reducing repeated cellular turnover in the ovaries [61].

#### 3.2.3. Endometrial Cancer

Endometrial cancer is the most prevalent type of gynecologic malignancy in women. Around 5% of all cancers and over 2% of cancer-related deaths are due to this cancer. In developed nations, it is the fourth most common cancer diagnosed in women [62]. It often presents with abnormal uterine bleeding, especially postmenopausal bleeding [60]. Endometrial cancer is a hormone-sensitive tumor, with 80% developing due to excess estrogen or low progesterone [47]. The recent findings establish a connection between endometrial cancer and a variety of elements, including genes, BMI, lifestyle habits, and medical issues like diabetes and PCOS. Factors that bring about prolonged estrogen exposure, for example, the number of parities, age at menarche, use of OCPs, etc., are associated with increased risk. Women who underwent menopause after the age of 46.5 years had a higher risk of endometrial cancer [62]. Excess estrogen has a mitogenic effect on endometrial cells, which can be caused by either endogenous or exogenous estrogen levels. Proliferating faster cells are more susceptible to errors in DNA replication, and the cells undergoing mutation can turn into cancer, usually adenocarcinomas. In a normal endometrium, the proliferative effects of estrogen are countered by progesterone, while the lack of progesterone post menopause leads to an unopposed effect of estrogen. Diets high in fat and high glycemic index diets promote obesity and insulin resistance, increasing the chances of developing endometrial cancer. High central obesity and hyperinsulinemia add to the effect by accelerating the proliferation of endometrial cells [47]. HRT use is also associated with endometrial cancer. Unopposed estrogen notably elevates the risk, while cyclic combined estrogen-progestin treatment largely reduces this risk [53].

#### 3.2.4. Vulvar and Vaginal Cancers

Vulvar cancer is most commonly diagnosed in postmenopausal women, with a median age at diagnosis of 65 years. Estrogen deficiency post-menopause leads to atrophic and dystrophic changes in the vulva, particularly lichen sclerosis. These chronic irritations and dystrophies are risk factors for the development of vulvar cancer. Vaginal cancer, on the other hand, is comparatively rare but also primarily affects postmenopausal women. Similarly to vulvar cancer, the atrophic vaginal state of vaginal tissues contributes to malignant transformation [60].

### 3.3. Osteoporosis and Fractures: Hip, Vertebral, and Fragility Fractures

Osteoporosis is one of the major and common complications in postmenopausal women, mainly caused by the drop in estrogen levels after menopause. An imbalance in bone remodeling causes osteoporosis, a systemic skeletal condition that reduces bone strength, disrupts microarchitecture, and makes the skeleton more brittle, increasing the risk of fracture [63]. The gold standard for diagnosing osteoporosis is dual X-ray absorptiometry (DXA), which measures bone mineral density (BMD) [63]. Estrogen has a protective effect on bone, and hormone therapy remains a key strategy in preventing bone loss in postmenopausal women [64]. Multiple studies, including large meta-analyses and randomized controlled trials like the HOPE WHI studies, have demonstrated that Menopause Hormone Therapy (MHT) significantly increases BMD at key sites such as the spine, hip, and forearm, and effectively reduces the risk of osteoporotic fractures [64]. Discontinuation of MHT leads to rapid bone loss, reversing much of the BMD gained and significantly increasing fracture risk. A study by Cummings SR et al., a randomized trial involving 4538 postmenopausal women at high risk of fracture, tibolone treatment led to significant improvements in BMD, with a 4.8% increase in the spine and a 3.1% increase in the femoral neck compared to placebo [65]. Tibolone is a synthetic steroid with estrogenic, progestogenic, and androgenic properties, and has shown effectiveness in reducing fracture risk in this population [65]. Although tibolone also showed a protective trend for hip fractures, the data were not statistically significant. Tibolone may help improve bone strength and reduce fracture risk, but its potential side effects, like stroke, should be considered carefully [65]. Osteoporosis in postmenopausal women is generally managed through both non-pharmacological and pharmacological strategies aimed at reducing fracture risk and improving bone health. Non-pharmacological measures include regular weight-bearing and resistance exercises to enhance strength and balance, adequate intake of calcium (1000–1500 mg/day) and vitamin D (600–800 IU/day) through diet or supplements is essential [66]. Pharmacological treatment is recommended for women with a history of fragility fractures, a T-score ≤ −2.5, or osteopenia with high fracture risk. MHT, which is commonly used, is effective for symptom relief and fracture prevention in younger postmenopausal women but is used cautiously in older women due to risks such as venous thromboembolism (VTE), stroke, and breast cancer [66]. In older or high-risk women, bisphosphonates are the first-line agents, effectively reducing various fractures, and are often followed by drug holidays. Denosumab, a biannual injection, is effective even in renal impairment but requires transition therapy upon discontinuation. Teriparatide, an anabolic agent, is reserved for those at very high risk, typically followed by antiresorptives to maintain gains. Treatment choices depend on age, fracture risk, comorbidities, and individual preferences [66].

### 3.4. Metabolic Disorders: Type 2 Diabetes, Lipid Abnormalities

Menopause induces a hormonal transition, affecting multiple systems, with an increase in the risk of metabolic disorders [67]. Menopause induces significant changes in body composition, lipid metabolism, and basal metabolic rate subsequent primarily due to the fall in estrogen levels and relative rise in androgens. This hormonal change leads to an increase in visceral fat accumulation with little change in BMI since hip and thigh fat redistributes to the abdomen [68]. These changes lead to central adiposity, insulin resistance, and alteration in lipid profiles, resulting in the development of metabolic syndrome (MetS) and non-alcoholic/metabolic-associated fatty liver disease (NAFLD/MAFLD). MetS, diagnosed based on diagnostic criteria like abdominal obesity, dyslipidemia, hypertension, and hyperglycemia, rises post-menopause. Pathophysiology of MetS involves inflammation mediated by visceral fat, endocrine derangement, and hypothalamic–pituitary–adrenal axis dysregulation [67]. Both aging and menopause contribute to metabolic risk, but there is evidence that menopause itself enhances susceptibility to these conditions [67]. Sarcopenia, characterized as skeletal muscle loss and weakness, is often present with MetS. Skeletal muscle is crucial for insulin-stimulated glucose metabolism, and the loss of skeletal muscle contributes to insulin resistance (IR) and metabolic disturbances [67,68]. Lipid metabolism is adversely affected, with rising levels of total cholesterol, LDL-C, and triglycerides and a higher ratio of total cholesterol to HDL during menopause transition. HDL-C levels are variable, but there is a shift towards smaller, less protective HDL particles [68]. Menopause is also marked by denser, smaller LDL particles, which are more atherogenic. All these metabolic changes enhance the risk of cardiovascular disease and underscore the need for targeted interventions in postmenopausal women [68]. Management of MetS in postmenopausal women depends on body weight and metabolic risk. Women with normal weight should focus on preventing weight gain, and overweight/obese women should focus on strategies required to lose weight and manage insulin resistance [69]. Lifestyle change, specifically a hypocaloric diet and daily physical exercise, improves body composition, lipid profile, and glucose metabolism. High-intensity interval training (HIIT) is more effective in reducing visceral fat. HRT, particularly transdermal estrogens, is useful in the prevention of undesirable changes in body composition in normally weighted women with menopausal symptoms. Lifestyle modification and HRT alone may not be sufficient to control overweight or obese women with metabolic syndrome (MS), particularly with insulin resistance [69]. Pharmacological interventions like metformin and using compounds like myo-inositol (MYO), D-chiro-inositol (DCI), alpha-lipoic acid (ALA), carnitines, and L-arginine can improve insulin sensitivity and help in weight management [69]. Since these metabolic vulnerabilities are genetically or epigenetically determined, and given the importance of personalized treatment during menopausal transition, a thorough clinical history is important.

## 4. Minor Complications

Though prior research revolves mostly around major complications of menopause, minor yet impactful changes in menopause are also significantly compromising the quality of life of postmenopausal women. A wide range of complications, including ophthalmic changes, genitourinary issues, sleep cycle disorders, mood alterations, and changes in intraocular and blood pressure regulation, are considered less severe but require clinical attention. We have evaluated scientific pieces of evidence for the association between menopause and somatic or psychological problems; the temporal association of different symptoms with the menopausal transition.

### 4.1. Vasomotor Symptoms

Approximately 75% to 85% of menopausal women experience vasomotor symptoms [70]. Vasomotor symptoms (VMS), including hot flashes and night sweats, are among the earliest and most common manifestations of menopause. The physiology involves estrogen withdrawal leading to hypothalamic thermoregulatory center instability, which reduces the thermoneutral zone. Minor core body temperature changes trigger sweating and flushing episodes. Studies, including Altura’s vascular research [71], indicate that the responsiveness of arterioles to catecholamines is greater in women with hot flushes than in those without hot flushes. Estrogen appears to enhance α2-adrenergic activity, and estrogen withdrawal may therefore lead to vasomotor flushes as a result of reduced α2-adrenergic activity.

### 4.2. Ocular Dysfunctions

Menopausal and postmenopausal women are especially at risk of “Dry Eye Syndrome” because of hormonal dysregulation of the secretory glands in the eyes. Estrogen deficiency contributes to ocular surface inflammation and structural changes in the conjunctival epithelium. Additionally, intraocular pressure tends to rise post-menopause, increasing the risk of glaucoma [72]. Estrogen has neuroprotective effects on the retina and helps regulate IOP and outflow resistance. Estrogen deficiency during menopause may contribute to glaucoma development by influencing intraocular pressure (IOP), retinal ganglion cell (RGC) survival, aqueous humor outflow resistance, and ocular biomechanical properties. Nearly 60% of postmenopausal women experience dry eye symptoms [73], and elevated intraocular pressure is notably higher in those with systemic hypertension [72].

### 4.3. Urological Dysfunctions

As the estrogen levels decrease during the peri menopausal period, the risk of UTIs increases. This is associated with impaired bladder emptying, dilatation of the urethra, decreased integrity of the urothelial epithelium, and disruption of the vaginal microbiome. Deteriorated bladder emptying causes urine retention, which then leads to bacterial proliferation and impaired bladder clearance. Estrogen deficiency results in disruption of the integrity of the urothelium by reducing the amount of E-cadherin linking proteins, Gębka et al. [74]. In addition, a decrease in estrogen levels causes a decrease in the level of glycogen, which is a substrate for lactic acid bacteria. The disruption of the natural bacterial flora of the vagina causes increased frequency of infection, and because the urethra is atrophied and dilated, it is much easier for infection from the vagina to spread to the urinary tract. During menopause, the decreased concentration of estrogen is responsible, among other causes, for the proper functioning of the epithelium of the inner surface of the urethra and the bladder. The urethra becomes dilated, resulting in involuntary urine leakage due to decreased resistance and the need for increased tension of the sphincters, whose tension decreases during menopause, causing urinary incontinence. Urinary incontinence affects 30% to 50% of postmenopausal women [74]. Up to 20% experience recurrent UTIs. Urinary symptoms cause embarrassment, social withdrawal, anxiety, and sexual dysfunction due to fear of incontinence during intercourse [74].

### 4.4. Nephrolithiasis

Estrogen deficiency alters calcium metabolism and changes urinary composition, including decreased citrate excretion and increased urinary calcium levels, promoting kidney stone formation. The incidence of nephrolithiasis increases by 25–35% in postmenopausal women compared to premenopausal peers [74]. Episodes cause severe flank pain, hospital admissions, procedural interventions, and disruption of daily activities.

### 4.5. Sexual Dysfunction

Sexual dysfunction during menopause involves vaginal atrophy, decreased lubrication, painful intercourse (dyspareunia), and loss of libido. Estrogen deprivation leads to vaginal tissue thinning, loss of elasticity, and reduced arousal response. Sexual complaints affect 50–75% of postmenopausal women [75], yet less than 2% seek or receive treatment [75]. Underreporting due to social stigma, lack of clinician screening, and fear of HRT side effects contributes to poor management. Sexual dysfunction leads to relationship stress, decreased intimacy, low self-esteem, and emotional distress.

### 4.6. Elevation in Blood Pressure

During and after menopause, although not always symptomatic, it reflects another subtle but important complication. Menopause-related hypertension is often insidious and undertreated, especially nocturnal hypertension. Mounier-Vehier and Madika [70] outlined how estrogen loss impairs vascular compliance, increases salt sensitivity, and promotes renal dysfunction, driving postmenopausal blood pressure elevation. They also pointed out that lifestyle factors such as obesity and high salt intake further exacerbate postmenopausal hypertension. Although thiazide diuretics are recommended as first-line agents, careful attention to early blood pressure monitoring and tailored lifestyle interventions is essential. In selected women with climacteric symptoms and no cardiovascular contraindications, HRT using transdermal 17β-estradiol may offer additional benefits by mitigating vascular stiffness and improving endothelial function [70].

### 4.7. Sleep Disturbance

Insomnia is one of the most common complaints among menopausal women. Proserpio et al. [76] applied the “3-P Model,” which includes predisposing (age-related insomnia risk), precipitating (hormonal shifts, VMS, pain), and perpetuating factors (maladaptive behaviors) to explain this phenomenon. Fluctuating estrogen levels disrupt melatonin secretion and circadian rhythm stability, contributing to fragmented sleep. Insomnia affects approximately 40–60% of postmenopausal women [76].

### 4.8. Mood Disorders

Menopause is associated with increased vulnerability to depression and anxiety disorders. Neurochemical changes involving serotonin, dopamine, and GABA systems, coupled with life-stage stressors, contribute to mood instability. The incidence of depressive symptoms increases by 15–20% during perimenopause and menopause [77].

### 4.9. Gastrointestinal Complications

**Figure 1:**
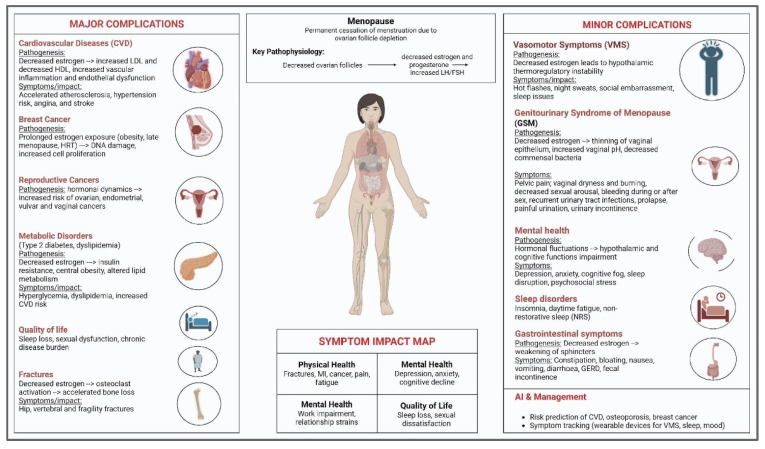
Overview of major and minor complications of menopause (created with BioRender.com, version 04).

Perimenopausal and postmenopausal women experience gastrointestinal (GI) symptoms, a serious but frequently overlooked range of issues that have a substantial impact on their quality of life and mental health [78]. Nausea, vomiting, diarrhea, stomach discomfort, constipation, bloating, gastroesophageal reflux, and fecal incontinence are frequent symptoms that can last for years following the onset of menopause [78]. Individuals and populations differ greatly in the frequency and intensity of gastrointestinal symptoms, which are impacted by lifestyle, genetics, nutrition, and access to healthcare. GI problems are often disregarded in typical menopausal care guidelines, despite their prevalence and significance. This represents a significant gap in the holistic management of women’s health [78]. Compared to their premenopausal counterparts, postmenopausal women experience noticeably more severe cases of irritable bowel syndrome (IBS). According to a study, postmenopausal women with IBS experienced worse physical health-related quality of life and more severe IBS symptoms than premenopausal women with IBS. Additionally, constipation is the main IBS subtype in women and rises with age for both sexes [79]. This shows that GI function and visceral sensitivity are impacted by brain–gut interactions that are modulated by lowering estrogen and progesterone levels throughout menopause [80]. New studies emphasize how important the gut microbiota is to menopausal gastrointestinal health. The diversity of the gut microbiota decreases with menopause, and its composition shifts toward that of men [81]. According to research, postmenopausal women’s gut microbiomes may become more like those of men because of a decrease in gut microbiome diversity brought on by decreased estrogen and progesterone after menopause. The distribution of gut microbiota in postmenopausal women varies depending on the level of follicle-stimulating hormone, with changes in gut microbiota abundance preceding symptom onset, according to one study that used 16S rRNA sequencing to identify differences in gut microbiota and metabolites among 44 women with menopausal syndrome [81]. Dysregulation of the digestive system is also caused by estrogen deficiency’s effects on the enteric nerve system, which controls motility and secretion. Through the brain–gut axis, psychological stress and mood disorders that are typical during menopause also impact GI function, influencing digestion, hunger, and visceral pain perception. Emotional stress can make GI symptoms worse by changing gut motility and cortisol levels, which makes digestion more difficult [21]. A comprehensive and interdisciplinary strategy is necessary to treat gastrointestinal issues in postmenopausal women. To address the intricate interactions between hormonal, metabolic, and psychosocial factors impacting GI symptoms, this involves dietary and lifestyle changes, tailored pharmaceutical and non-pharmacologic therapies, and psychological support. To better identify and treat these frequently incapacitating symptoms and enhance postmenopausal women’s general health and quality of life, healthcare professionals must become more knowledgeable and build individualized management plans [78,79]. Figure 1 shows an overview of major and minor complications of menopause with underlying pathophysiology, key symptoms, and impact on quality of life. Includes cardiovascular, oncologic, metabolic, skeletal, vasomotor, genitourinary, mental health, sleep, and gastrointestinal effects.

## 5. Artificial Intelligence (AI) in Risk Prediction

This section provides a thematic synthesis of recent literature on AI applications in postmenopausal health, consistent with a narrative-review approach. It integrates representative advances and interprets their clinical and translational relevance across major and minor complications. Collectively, these studies demonstrate how AI-driven technologies are reshaping risk prediction and personalized prevention in postmenopausal care.

### 5.1. AI Models Analyzing Biomarkers for Cardiovascular Disease and Osteoporosis

The postmenopausal decline in estrogen profoundly affects lipid metabolism and bone remodeling, predisposing women to cardiovascular disease (CVD) and osteoporosis. Machine-learning algorithms can analyze large datasets of lipid profiles, inflammatory markers, and bone-turnover indices to detect early pathological trends. Supervised models using HDL, LDL, and CRP values have stratified women by future CVD risk, enabling targeted lifestyle or pharmacologic interventions [82]. Similar frameworks assessing bone-metabolism biomarkers forecast fracture susceptibility and bone-mass loss. These biomarker-based models reveal AI’s strength in detecting subclinical metabolic and skeletal alterations, though most are trained on limited cohorts and lack standardized inputs for reproducibility.

### 5.2. Imaging AI for Mammography and DEXA Scans

AI-enhanced imaging has advanced early detection of breast cancer and osteoporosis—two major postmenopausal concerns. Deep-learning algorithms analyzing mammograms identify microcalcifications and masses that may escape visual detection, supporting screening, staging, and response prediction [83]. Applied to dual-energy X-ray absorptiometry (DEXA), AI improves precision in bone-density interpretation, quantifies subtle cortical changes, and predicts fracture risk by integrating demographic and clinical data. Imaging-based AI is among the most mature applications in this field, though wider adoption depends on validation across population-diverse datasets and regulatory approval of algorithmic outputs.

### 5.3. Metabolic-Syndrome Risk Prediction

Postmenopausal women are at heightened risk for metabolic syndrome, encompassing type 2 diabetes, obesity, hypertension, and non-alcoholic fatty-liver disease. AI systems combining fasting glucose, BMI, liver-function tests, and waist-to-hip ratio can synthesize longitudinal health-record data to predict disease onset before clinical thresholds [84]. These models highlight AI’s capacity to merge heterogeneous metabolic data for preventive care, but external validation across ethnic and socioeconomic groups remains limited.

### 5.4. Identifying Minor Complications

#### 5.4.1. Wearable Sensors and AI for Tracking Hot Flashes, Sleep, and Mood

Wearable sensors coupled with AI continuously monitor physiologic parameters—skin temperature, heart rate, and galvanic-skin response—to identify vasomotor instability, disordered sleep, and mood fluctuations. Algorithms analyzing these signals detect patterns of hot-flash frequency, reduced REM sleep, or heart-rate variability associated with anxiety or depression [85]. This continuous feedback shifts menopause management from episodic reporting to proactive monitoring, though accuracy still depends on device calibration and sustained user engagement.

#### 5.4.2. NLP Tools Mining EHRs and Patient Journals for Underreported Symptoms

Natural-language-processing (NLP) tools extract structured insights from unstructured clinical text and patient diaries, helping uncover underreported symptoms such as vaginal dryness, urinary urgency, and low libido [86,87]. By mining coded and free-text data, NLP expands awareness of genitourinary syndrome of menopause and strengthens patient-reported-outcome tracking. The approach, however, demands rigorous privacy protection and contextual validation before large-scale clinical use.

#### 5.4.3. AI for Cognitive Decline and Alzheimer’s Risk

Multimodal AI frameworks combining neuroimaging, cognitive testing, and genomic data are increasingly applied to predict cognitive decline and Alzheimer’s risk among postmenopausal women [88]. These models detect subtle cortical and metabolic changes years before clinical onset, offering opportunities for neuroprotective intervention. Broader implementation will require longitudinal datasets and clear ethical standards for neurodata handling.

#### 5.4.4. Pharmacogenomics for Hormone-Therapy Response Prediction

AI is also driving progress in pharmacogenomic modeling of hormone-replacement therapy (HRT). Machine-learning algorithms analyzing genomic variants can forecast treatment efficacy and side-effect likelihood, guiding regimen selection and reducing adverse outcomes such as elevated breast-cancer risk [89]. Personalized-therapy prediction represents a key step toward precision medicine, though integration with clinical-decision systems and multicenter genomic validation remain necessary for routine practice.

### 5.5. Comparative Summary and Emerging Directions

**Figure 2:**
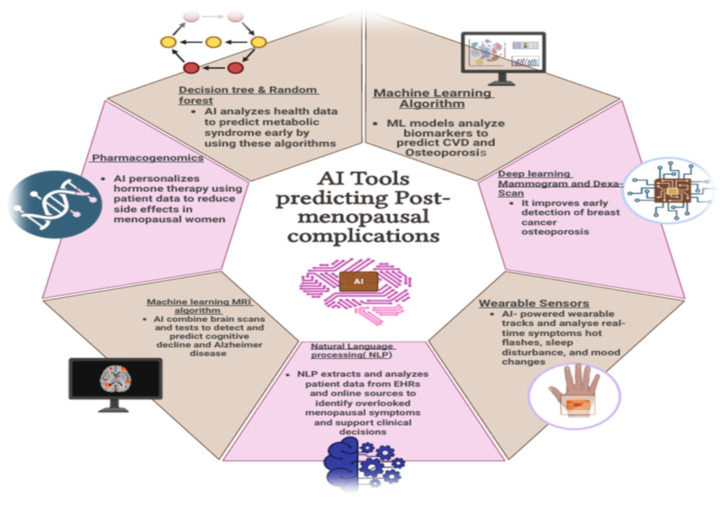
AI technologies for early detection, risk prediction, and personalized management (created with BioRender.com).

Across these domains, imaging-based and wearable-sensor AI applications are advancing most rapidly, benefiting from well-defined datasets and measurable outcomes. NLP and pharmacogenomic approaches remain earlier in development, constrained by privacy, interpretability, and interoperability challenges. The main bottlenecks include limited external validation, algorithmic bias, and inadequate integration with electronic health-record systems. Future progress will depend on federated-learning collaborations, transparent model reporting, and clinician education. Addressing these priorities will accelerate AI’s transition from experimental prediction to practical, preventive, and patient-centered care in menopause (Figure 2 and Table 1).

## 6. Discussion

Artificial intelligence (AI) has progressed from an educational adjunct into a transformative force redefining how postmenopausal complications are predicted, prevented, and managed. Early innovations such as the Artificial Intelligence-Based Program (AIBP) [92] illustrated how adaptive, digital education improved preventive awareness and lifestyle modification. Yet the same computational frameworks now drive diagnostic and therapeutic advances, connecting physiological, behavioral, and environmental determinants of women’s health. By integrating data from wearable devices, imaging, and genomics, AI enables a holistic and continuous approach to care; shifting the clinical paradigm from reactive treatment to proactive precision health.

### 6.1. AI in Managing Major Complications

The application of AI to major postmenopausal morbidities demonstrates its capacity to merge predictive diagnostics with individualized management. Cardiovascular disease (CVD), a principal source of morbidity after menopause, has benefited from early AI integration. Machine-learning and deep-learning algorithms can analyze complex multimodal inputs—electrocardiograms, echocardiograms, laboratory data, and clinical histories—to identify subclinical risk patterns long before symptoms appear [93]. Ensemble modeling, which integrates multiple predictive algorithms, increases model stability, and minimizes bias, allowing dynamic and personalized risk estimation rather than static scoring. Such adaptive surveillance models expand traditional prevention strategies by continuously updating risk profiles as patient variables evolve.

Comparable innovation is evident in oncology and skeletal health. In endometrial and breast cancers, AI systems incorporating clinical variables and imaging markers such as age, body mass index, and endometrial thickness achieve high discrimination for early disease [94,95]. Neural-network classifiers enhance recognition of subtle morphologic abnormalities, while convolutional networks (CNNs) extract high-resolution features from mammograms and pelvic ultrasonography to facilitate earlier and more accurate triage [96]. In osteoporosis, AI algorithms have surpassed conventional tools like FRAX by combining micro architectural, metabolic, and genetic data [97]. Whittier et al. [98] showed that clustering of HR-pQCT images identified distinct bone phenotypes predictive of fracture risk, emphasizing AI’s ability to capture biological diversity invisible to two-dimensional densitometry. Together, these studies signal a transition toward precision risk assessment in which AI augments clinician judgment, transforming fragmented diagnostics into integrated, data-driven prevention.

### 6.2. AI Managing Minor Complications and Quality of Life Domains

AI technologies are equally influential in addressing non-lethal yet life-altering postmenopausal symptoms. Intelligent conversational agents such as MenoBot [99] exemplify patient-centered design, merging wearable data with self-reported information on sleep, mood, and vasomotor activity. Through adaptive feedback loops, these systems provide personalized recommendations for hormonal therapy, nutrition, and behavioral adjustment, aligning day-to-day management with symptom variation.

Objective monitoring has particularly advanced vasomotor symptom detection: algorithms analyzing sternal skin conductance have achieved sensitivities above 80% and specificities approaching 97%, enabling real-time assessment and individualized therapy [9,14,100]. AI-assisted interpretation of pelvic-floor and urogenital parameters similarly supports early detection of estrogen-related tissue changes and optimizes hormone-replacement timing.

Sleep and cognitive disturbances—among the most common and distressing sequelae of menopause—are now being targeted through AI-enhanced behavioral frameworks. Machine-learning-driven cognitive behavioral therapy (AI-CBT) platforms personalize interventions according to neurocognitive profiles, extending evidence-based therapy to populations with limited specialist access [101]. AI tools assessing subjective cognitive impairment integrate psychological, metabolic, and lifestyle variables to flag early decline and its modifiable risk factors [100]. Collectively, these innovations redefine symptom management as continuous, data-supported care, linking physiologic insight with daily well-being and setting the foundation for the broader critical reflections discussed below.

### 6.3. Critical Analysis and Future Directions

Across current studies, AI appears technically advanced but clinically premature. Many models report excellent performance metrics—often exceeding 0.9 AUC—yet remain constrained by limited datasets, lack of external validation, and variable interpretability. The AIBP [92] improved health literacy but depended on user engagement and reliable connectivity, limiting scalability. MenoBot [99] illustrates the tension between personalization and data accuracy, as reliance on self-reported symptoms introduces recall bias and undermines clinical robustness. In cardiovascular risk modeling, deep-learning systems detect subclinical disease patterns with remarkable granularity [93]; however, their training on retrospective, demographically narrow datasets raises the risk of over fitting and reduced generalizability. Similarly, endometrial-cancer predictors [95] show near-perfect accuracy but remain untested across imaging modalities and ethnic groups. CNN-based imaging tools [96] enhance diagnostic sensitivity yet operate as “black boxes,” obscuring how predictions are derived—an issue that complicates clinical trust and regulatory approval. Osteoporosis algorithms [97,98] uncover novel bone phenotypes but rely on small, highly selected cohorts. Translating these insights into community screening will require affordable imaging, transparent model interpretation, and large-scale longitudinal data. Gynecologic AI such as the PSO-RF model for ovarian endometrioma [102] highlights computational ingenuity yet extrapolates from premenopausal datasets, demonstrating the need for hormonal-context specificity. Within quality-of-life domains, biosensor-based hot-flash detection [9,14,100] and AI-CBT for insomnia [101] demonstrate feasibility but lack standardized outcome measures and long-term validation. Overall, innovation continues to outpace implementation, with few models yet tested in prospective clinical environments.

Data heterogeneity further complicates reproducibility. As Díaz et al. [103] note, the absence of standardized, demographically diverse datasets undermines external validity. In low- and middle-income countries, algorithmic imports without contextual calibration risk amplifying inequities [104,105]. Ethical implementation thus requires not only transparent algorithms but also culturally responsive governance and robust health-system support. Future progress depends on establishing validated, interpretable, and equitable AI ecosystems. Multicenter collaborations should develop open-access menopause datasets encompassing genetic, behavioral, and environmental dimensions to ensure inclusivity. Explainable-AI (XAI) frameworks can clarify decision logic, strengthening both clinician confidence and patient autonomy. Integration with electronic health records through standardized protocols will create adaptive learning cycles where algorithms refine predictions as new outcomes emerge. In resource-limited settings, lightweight, offline-capable tools with fair data-governance policies can enhance accessibility without deepening digital divides. Finally, embedding AI literacy into medical curricula will empower clinicians to evaluate, rather than merely adopt, algorithmic outputs.

Emerging AI methodologies further illustrate how these challenges might be addressed. Human–AI hybrid systems such as the Pathology Expertise Acquisition Network (PEAN) have reduced manual annotation workloads by nearly 96% through computational use of pathologists’ eye-tracking data, demonstrating how human expertise can be scaled efficiently [106]. Similarly, the scEMC framework integrates multimodal datasets such as scRNA-seq and ATAC-seq through skip-aggregation networks to identify biologically robust patterns [107]. Complementary approaches using high-order topological fuzzy clustering capture nonlinear, multiview relationships across heterogeneous data structures [108]. Although not yet applied directly to menopause research, these strategies exemplify transferable solutions for managing heterogeneity, multimodal integration, and interpretability—issues central to advancing AI in women’s health. In conclusion, AI in postmenopausal health has reached computational sophistication but not yet clinical maturity. Its next leap will rely less on higher accuracy and more on reproducibility, explainability, and ethical inclusivity [109]. When these standards are met, AI will transition from a promising adjunct to a reliable partner in improving the health and quality of life of women worldwide.

### 6.4. Practical Integration with Healthcare Systems

While AI in postmenopausal health has demonstrated encouraging outcomes in research environments, its true value will be realized only when these innovations are embedded within healthcare systems and scaled equitably across diverse populations. Bridging the gap between algorithmic performance and clinical adoption requires practical alignment with workflows, infrastructure, and policy frameworks. Embedding AI models within electronic health records (EHRs) and clinical decision-support systems enables clinicians to receive timely, context-specific recommendations during patient encounters. When models combine EHR data with patient-generated inputs from wearable devices or mobile applications—such as sleep, vasomotor, or mood-tracking logs; predictions become more individualized and clinically relevant. Interoperability standards, transparent outputs, and reduced clinician workload have emerged as critical determinants of adoption [110,111].

**Figure 3:**
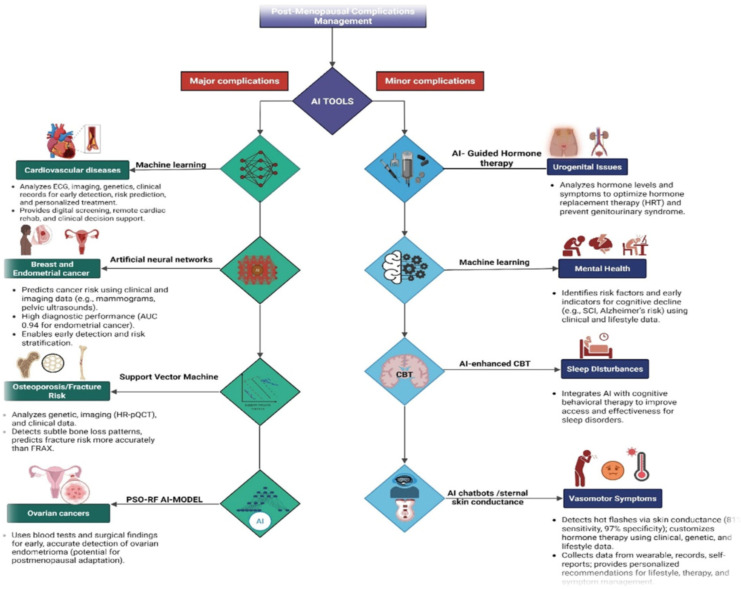
AI-driven approaches for managing postmenopausal complications (created with BioRender.com).

AI-enhanced telemedicine also offers a pragmatic avenue to extend specialist care to regions where medical expertise is scarce. Integrating AI into telehealth platforms facilitates remote monitoring, triage, and diagnostics, provided that barriers related to connectivity, affordability, and digital literacy are addressed [112,113]. Evidence from rural health initiatives cautions that without local validation, culturally adapted design, and offline-capable architecture, these systems risk reinforcing disparities rather than reducing them [114]. Ensuring inclusion of diverse socio-demographic groups in model development remains essential to equitable implementation. Sustainable integration also depends on governance structures that maintain clinician trust and patient safety. Deployed algorithms must be monitored for bias, model drift, and unintended consequences. Implementation frameworks such as RE-AIM (Reach, Effectiveness, Adoption, Implementation, and Maintenance) offer structured methods to evaluate performance, equity, and scalability across settings [115,116,117]. Real-world validation through iterative feedback loops can ensure that AI systems continue to perform reliably under dynamic clinical conditions. A pragmatic pathway for postmenopausal AI integration therefore involves embedding validated risk models into EHRs, linking predictive outputs to telehealth follow-up workflows, and adapting digital platforms for rural contexts through offline or mobile capabilities and culturally tailored design. Continuous evaluation of performance and equity impacts using established implementation frameworks will be vital to ensure that AI complements existing healthcare infrastructure rather than disrupts it. Figure 3 highlights AI driven approaches for managing postmenopausal complications, showing how machine learning and related tools enables detection and personalized care for both major and minor complications.

## 7. Conclusions

Artificial intelligence is rapidly redefining postmenopausal healthcare by enabling earlier prediction, refined diagnosis, and truly personalized intervention. Across cardiovascular, skeletal, oncologic, and mental-health domains, AI-driven models are uncovering subtle biological and behavioral patterns that were previously invisible to clinicians. Deep learning, natural-language processing, and multimodal data fusion are moving menopause management from reactive treatment to proactive, precision-based care. Yet, clinical adoption remains uneven, and the gap between research innovation and bedside application continues to challenge meaningful translation. A central obstacle lies in bridging research and real-world practice. Most current studies rely on retrospective or single-center datasets with limited demographic diversity. To achieve generalizable performance, collaborative, multicentric databases representing the full spectrum of postmenopausal women are essential. Equally important is model explainability—clinicians must understand why an algorithm makes a prediction before trusting it in decision-making. Ethical, legal, and social considerations must advance in parallel. Because AI models frequently handle sensitive reproductive, psychological, and genetic information, transparent consent, strong data-security protocols, and equitable access policies are imperative. Algorithmic bias remains a critical concern, underscoring the need for fairness metrics and inclusive datasets that prevent amplification of gender or racial disparities.

Looking ahead, the convergence of AI with precision-medicine principles will shape the future of menopausal care. Wearable sensors, digital biomarkers, and telehealth platforms can continuously monitor physiologic and behavioral signals, allowing predictive algorithms to identify vasomotor, metabolic, or cardiovascular risks before clinical onset. Adaptive learning systems—supported by responsive regulatory frameworks—can maintain safety while updating with new data. At the same time, success depends on education and collaboration: clinicians require literacy in data analytics, data scientists need deeper understanding of hormonal physiology, and women themselves must be empowered to engage with AI tools confidently. In the coming decade, AI’s role will expand from supportive decision-making to integrated clinical reasoning. Multimodal models synthesizing imaging, genomic, and lifestyle data may redefine risk assessment, while conversational agents could enhance symptom tracking and emotional well-being. The trajectory of innovation points toward a holistic framework that unites biological, psychological, and environmental dimensions of menopause. Ultimately, AI’s promise will be realized only through transparent governance, ethical stewardship, and a sustained commitment to inclusivity. By aligning algorithmic design with women-centered values and clinical evidence, AI can transform menopause care from episodic management into an anticipatory, data-driven continuum of lifelong wellness [22,118,119].

## Figures and Tables

**Table 1:** Different AI Models Used for the Prediction of Post-Menopausal Complications.

Year	Author/Study	Technique	Results and Limitations
**2023**	Ahn et al. [83]	Review of AI in breast cancer diagnosis and personalized treatment	AI shows promise in screening, staging, and treatment prediction, especially in imaging and pathology. Limitations include clinical validation needs, model generalizability, and integration challenges.
**2025**	Carlson & Nguyen [86]	Educational Review on GSM	Discusses hormonal changes and symptoms during menopause and outlines treatment options. Limitations include underdiagnosis and hesitancy among women to seek care.
**2024**	Hu et al. [82]	Unsupervised ML clustering using EMR data (K-means, PCA)	Achieved predictive accuracy > 85% in identifying CVD cases. Limitations include a lack of longitudinal design and a need for further refinement for clinical use.
**2024**	Khatiwada et al. [87]	Systematic review using PRISMA and Rayyan on PGHD	Identified key challenges in PGHD, including privacy, security, and stakeholder understanding. Limitations include fragmented standards and a lack of regulatory clarity.
**2023**	Ong et al. [90]	Systematic review of AI classification of osteoporosis via CT	AI achieved accuracy between 61.8 and 99.4% using CT scans. Limitations involve variability in methods, need for validation, and comparison with the DEXA gold standard.
**2023**	Davis et al. [91]	Scientific review on menopause biology and treatment	MHT and non-hormonal treatments are effective; personalized care is emphasized. Limitations include a lack of data on perimenopausal women and treatment safety.

## Data Availability

The review was based on publicly available academic literature databases.
